# 

**Published:** 1890-02

**Authors:** 


FEBRUARY, 1890.

LIB[illegible] Inde[illegible] S. G.

### THE AMERICAN JOURNAL OF DENTAL SCIENCE.

EDITED BY

F. J. S. GORGAS, M. D., D. D. S.

Vol. 23. THIRD SERIES. No. 10.

BALTIMORE:
SNOWDEN & COWMAN, 9 W. Fayette Street.

LONDON:
TRUBNER & CO., 60 Paternoster Row.

PUBLISHED MONTHLY.

$2.50 PER ANNUM.

PAYABLE IN ADVANCE.

